# Assessment of Chemo-Immunotherapy Regimens in Patients with Refractory or Relapsed Neuroblastoma: A Systematic Review with Meta-Analysis of Critical Oncological Outcomes

**DOI:** 10.3390/jcm14030934

**Published:** 2025-01-31

**Authors:** Nur Olgun, Mehmet Emin Arayici, Deniz Kızmazoglu, Refik Emre Cecen

**Affiliations:** 1 Department of Pediatric Oncology, Institute of Oncology, Dokuz Eylul University, İzmir 35340, Turkey; deniz.kizmazoglu@deu.edu.tr (D.K.); rececen73@gmail.com (R.E.C.); 2 Acıbadem Kent Hospital, Karsiyaka, İzmir 35630, Turkey; 3 Department of Biostatistics and Medical Informatics, Faculty of Medicine, Dokuz Eylul University, İzmir 35340, Turkey; mehmet.e.arayici@gmail.com; 4 Department of Public Health, Faculty of Medicine, Dokuz Eylul University, İzmir 35340, Turkey

**Keywords:** meta-analysis, refractory, relapsed, neuroblastoma

## Abstract

**Background:** Neuroblastoma is a highly aggressive pediatric cancer, particularly in children with refractory or relapsed disease, where survival outcomes remain poor despite advancements in treatment. Combining anti-GD2 antibodies, such as dinutixumab beta, dinutixumab, and naxitanab, with conventional chemotherapy has emerged as a promising approach to improve clinical outcomes in this high-risk population. This chemo-immunotherapy regimen meta-analysis aimed to investigate the efficacy of these combination regimens by analyzing objective response rate (ORR), overall survival (OS), and event-free survival (EFS) outcomes across multiple studies. **Methods:** A systematic review and meta-analysis were conducted following PRISMA guidelines. PubMed, Web of Science, and Scopus databases were searched, yielding studies comprising the related reports. Both randomized controlled trials and non-randomized studies were included. The primary outcome of interest was ORR, and the secondary outcome of interest was EFS. A random-effects model using the DerSimonian–Laird method and Knapp–Hartung–Sidik–Jonkman adjustments was employed to pool effect sizes, and heterogeneity was assessed using I^2^ statistics. **Results:** A total of ten reports from eight studies were deemed eligible and included in the meta-analysis. The pooled ORR across the studies was 0.45 (95% CI: 0.35–0.54, *p* < 0.001), indicating that approximately 45% of patients showed a favorable treatment response, with moderate heterogeneity (I^2^ = 52.78%). The pooled analysis showed an OS of 75% (95% CI: 53–96, *p* < 0.001), and the pooled EFS effect size was 0.59 (95% CI: 0.45–0.73, *p* < 0.001), despite substantial heterogeneity (I^2^ = 60.54%). **Conclusions:** anti-GD2 antibodies combined with conventional chemotherapy may significantly improve response rates and event-free survival in children with refractory or relapsed neuroblastoma. Future research should focus on identifying predictive biomarkers to tailor therapies to individual patients, enhancing both efficacy and safety in this vulnerable population.

## 1. Introduction

Neuroblastoma is one of the most common extracranial solid tumors in children, accounting for approximately 8–10% of all childhood cancers and responsible for a significant proportion of cancer-related mortality in this population [1,2,3,4,5,6]. Despite advances in multimodal therapy, including surgery, chemotherapy, radiotherapy, and stem cell transplantation, outcomes for children with refractory or relapsed neuroblastoma remain dismal, with survival rates well below 50% [2,7]. The heterogeneous nature of neuroblastoma, both in its genetic profile and clinical behavior, has presented substantial challenges in achieving effective long-term remission, especially for high-risk patients. Despite multimodal therapy approaches, relapse or refractory disease is a problem, especially for high-risk patients, so there is an urgent need to explore novel therapeutic strategies that can improve outcomes.

Recent advances in molecular oncology have led to the development of targeted therapies that aim to disrupt specific pathways involved in the pathogenesis and progression of neuroblastoma. These therapies, which include agents targeting ALK mutations [8], GD2 [9], and MYCN amplification [10], offer the potential for more personalized treatment approaches, potentially improving outcomes when used in conjunction with conventional chemotherapy. Anti-GD2 antibodies are designed to enhance the efficacy of existing treatment regimens while minimizing toxicity, addressing the limitations of traditional chemotherapy, which often fails to eradicate the disease in refractory or relapsed cases [2,11].

Despite advances in neuroblastoma treatment, particularly for high-risk and relapsed cases, the clinical outcomes remain suboptimal, necessitating the exploration of novel therapeutic approaches. Several key studies have previously examined the efficacy of anti-GD2 antibodies, such as dinutuximab beta, irinotecan, and temozolomide, either as single agents or combined with conventional chemotherapy [5,7,12]. These studies, often conducted as primary trials, have demonstrated promising results in terms of objective response rates (ORR), event-free survival (EFS), and overall survival (OS) in pediatric patients with refractory or relapsed neuroblastoma. However, the heterogeneity in treatment protocols, patient populations, and outcomes across these individual trials has made it difficult to derive definitive conclusions. Although individual randomized and non-randomized studies have reported encouraging results, there is a growing need for a comprehensive synthesis of these data and to collectively evaluate the overall framework, particularly focusing on chemo-immunotherapy in refractory or relapsed neuroblastoma. Previous primary studies have primarily evaluated the use of single-agent immunotherapies or conventional chemotherapy alone, or they have included limited data on combination strategies. By systematically collating and analyzing all available evidence on chemo-immunotherapy, we aim to clarify the relative efficacy of these combinations and fill a critical gap in the existing literature. A meta-analysis focusing on combination regimens will allow for a more accurate assessment of the collective impact of chemo-immunotherapy in pediatric neuroblastoma. Therefore, there is a critical need to synthesize these data through a comprehensive meta-analysis to provide a more robust understanding of the collective efficacy of these regimens.

In this meta-analysis, we aim to critically evaluate the oncological outcomes of combining anti-GD2 antibody therapies with conventional chemotherapy in children with refractory or relapsed neuroblastoma. Our primary outcome of interest is the ORR, which provides a direct measure of the efficacy of these combination therapies in inducing tumor regression. Additionally, our secondary outcomes of interest include OS and EFS, both of which are crucial indicators of long-term treatment success and relapse prevention. By analyzing these key outcomes, we seek to provide evidence-based recommendations for the integration of anti-GD2 antibodies into standard treatment protocols. This meta-analysis will address the current gaps in knowledge and highlight the potential benefits and challenges associated with these combination therapies in improving outcomes for neuroblastoma patients facing this aggressive disease.

## 2. Methods

We performed this meta-analysis following the guidelines of the “Preferred Reporting Items for Systematic Reviews and Meta-Analyses (PRISMA)” statement [13], ensuring a thorough evaluation of the data available in the literature. The PRISMA checklist, which details the criteria and methodology used in the analysis, is provided in Supplementary Table S1. The study protocol was not recorded in an official register.

### 2.1. Literature Search and Data Sources

The data for this study were collected by two investigators (MEA and NO), who performed a comprehensive search of the literature using databases such as PubMed/Medline, Web of Science, and Scopus. To ensure the inclusion of the most up-to-date information, the search covered studies published up to 18 August 2024. The search strategy was carefully designed to maximize the retrieval of relevant studies, utilizing Medical Subject Headings (MeSH), keywords, text terms, and Boolean operators (AND/OR). The keywords were meticulously chosen to specifically focus on the targeted research area.

The primary keywords used in the search strategy included: ((((relapsed[Title/Abstract]) OR (refractory[Title/Abstract])) AND (neuroblastoma[Title/Abstract])) AND (((immunotherapy[Title/Abstract]) OR (chemotherapy[Title/Abstract])) OR (chemo-immunotherapy[Title/Abstract]))) AND (((((dinutuximab[Title/Abstract]) OR (dinutuximab beta[Title/Abstract])) OR (naxitamab[Title/Abstract])) OR (irinotecan[Title/Abstract])) OR (temozolomide[Title/Abstract])). The literature searches were conducted exclusively in English, with publications in other languages excluded from our search strategy. To present a clear and systematic overview of the search strategies applied to each relevant database, a detailed, structured form is included in Supplementary Table S2.

### 2.2. Study Selection Process

During the initial stage of the search process, two researchers independently reviewed the titles and abstracts of the identified studies. At this point, any papers that were deemed irrelevant or unsuitable for the study were promptly excluded. For further analysis, we selected studies focusing on anti-GD2 antibodies plus conventional chemotherapy in children with refractory or relapsed neuroblastoma. To ensure consistency in the dataset, clear definitions for “relapsed” and “refractory” neuroblastoma were applied during study selection. Relapsed neuroblastoma was defined as the recurrence of disease following an initial response to therapy, characterized by either progressive disease on imaging or a rise in relevant biomarkers, as described in prior consensus guidelines [14,15]. Refractory neuroblastoma was defined as the failure to achieve complete or partial response after at least one line of standard treatment, including chemotherapy, as outlined by previous studies and clinical criteria [14,15]. Only studies explicitly reporting on these patient populations and adhering to these definitions were included. To maintain the focus and relevance of our analysis, we excluded certain types of studies from this research. Specifically, non-original publications (e.g., letters to the editor) and studies that did not concentrate on relapsed/refractory were removed from consideration. To ensure the integrity of the data, a systematic approach was employed. Initially, we used the Mendeley data management tool to identify and remove any duplicate articles from the relevant databases, streamlining the dataset and preventing redundant results. Next, two independent authors meticulously reviewed the findings from each study, carefully extracting and synthesizing the necessary information. The data were then organized and consistently recorded in a pre-structured Microsoft Excel^®^ spreadsheet.

### 2.3. Data Extraction

In this meta-analysis, a thorough data extraction process was conducted for all eligible studies. This involved gathering pertinent details such as study characteristics, sample size, the country where the research was carried out, intervention specifics, follow-up duration, and other relevant information. In cases where discrepancies arose between the authors during data extraction, open communication and discussion were prioritized to resolve any differences and ensure accuracy.

### 2.4. Quality Assessment

In this study, the quality assessment of randomized controlled trials (RCTs) was performed using the RoB 2 tool [16], and non-randomized studies were evaluated using the ROBINS-I tool [17]. The RoB 2 tool assesses potential biases across five domains: bias arising from the randomization process, bias due to deviations from intended interventions, bias due to missing outcome data, bias in the measurement of the outcome, and bias in the selection of the reported result. For non-randomized studies, the ROBINS-I tool evaluates seven domains of bias: bias due to confounding, bias in the selection of participants, bias in the classification of interventions, bias due to deviations from intended interventions, bias due to missing data, bias in the measurement of outcomes, and bias in the selection of the reported result.

### 2.5. Statistical Analysis

The analyses were conducted using two meta-analytic approaches. To perform the meta-analysis, a Freeman-Tukey double arcsine transformation was applied to stabilize the variances of the pooled estimates. A random effects model, based on DerSimonian and Laird’s method with inverse variance, was employed for this purpose. Additionally, the Hartung–Knapp–Sidik–Jonkman method was used to adjust the standard error in the meta-analysis for the overall effect size [18,19]. Also, prediction interval values were calculated for the overall effect size. The choice of a random-effects model was based on the anticipated clinical and methodological heterogeneity across the included studies, reflecting differences in patient populations, treatment protocols, and outcome assessments. To address this variability, the Hartung–Knapp adjustments were applied to provide more accurate confidence intervals, particularly in the presence of small study effects or moderate heterogeneity. This approach ensured a robust synthesis of the data while accounting for both within- and between-study variability, enhancing the reliability of the pooled estimates. To assess the heterogeneity of the trials included in this study, we employed two well-established statistical methods: the χ2-based Cochran’s Q test and the I^2^ statistic. Significant heterogeneity was considered present when the Cochran’s Q test produced a *p*-value below 0.05 and the I^2^ statistic exceeded 50%. In the meta-analysis, effect sizes for objective response rate (ORR), one-year overall survival (OS), and event-free survival (EFS) were calculated, with 95% confidence intervals (CIs) used to quantify the associations between these outcomes. Sensitivity analyses were conducted by excluding all studies separately for ORR and EFS. Thus, the primary effect of each study on the overall outcome was evaluated in detail. Funnel plots for pooled analyses were also created. We operated under the assumption that the included studies originated from different samples and therefore applied the random effects model to all analyses. Meta-analysis statistical computations were carried out via STATA 18.0 (v18, College Station, TX, USA) [20]. In all tests conducted, a two-tailed *p*-value of less than 0.05 was set as the threshold for determining statistical significance.

## 3. Results

### 3.1. Study Selection Process and Quality Assessment of the Included Studies

**Figure 1:**
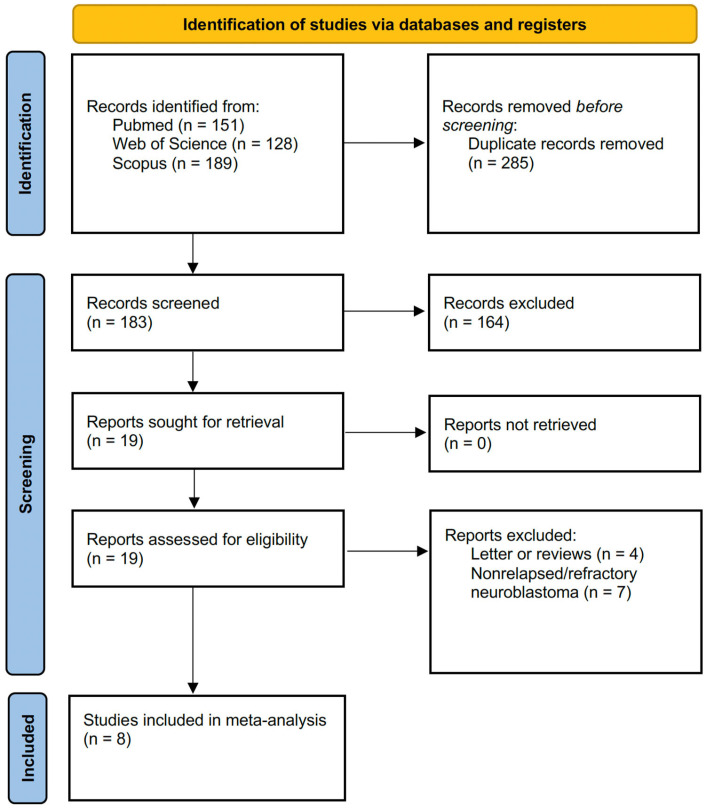
PRISMA flowchart showing summary of search strategy.

Initially, a total of 468 records were identified through comprehensive searches of three databases: PubMed (*n* = 151), Web of Science (*n* = 128), and Scopus (*n* = 189). After removing 285 duplicate records, 183 records were screened based on their titles and abstracts. Following this screening phase, 164 records were excluded due to irrelevance or failure to meet the inclusion criteria. This left 19 reports for further retrieval and eligibility assessment. No reports were excluded at this stage due to retrieval issues. Of the 19 reports assessed for eligibility, 13 were excluded, comprising four letters or review papers and seven studies that did not focus on relapsed or refractory neuroblastoma. Ultimately, eight studies [5,6,7,12,21,22,23,24] were deemed eligible and included in the final meta-analysis. Figure 1 illustrates the PRISMA flow diagram, offering a visual summary of the comprehensive database search and study selection process. Moreover, 256 registered trials regarding refractory or relapsed neuroblastoma were identified at https://clinicaltrials.gov (accessed on 24 January 2025). Additional information about the trials is presented in Supplementary Materials S1.

**Figure 2:**
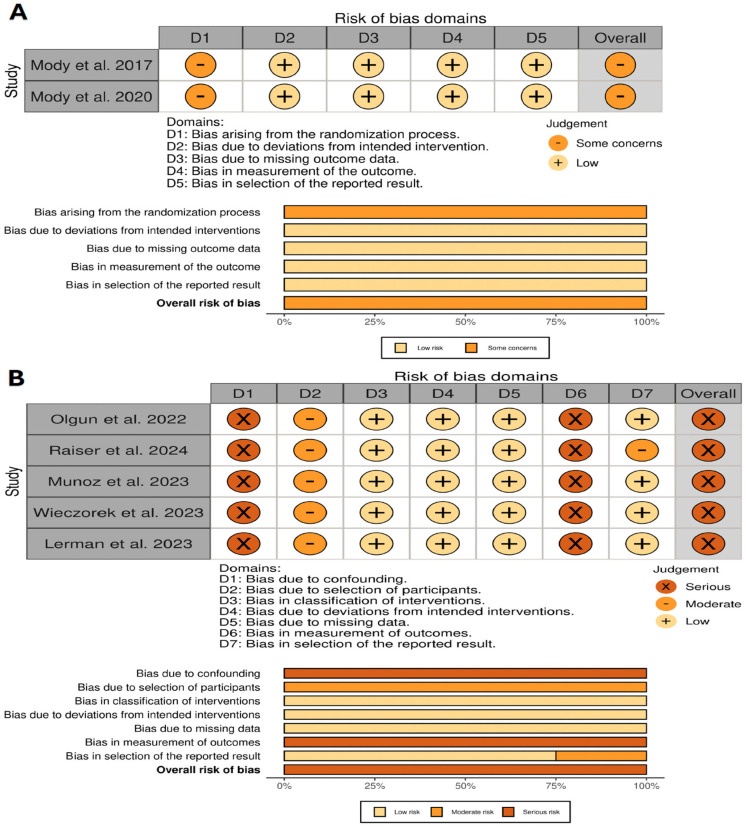
Risk of bias assessment of the included research. (**A**) RoB 2 tool [6,21]; (**B**) ROBINS-I tool [5,7,12,22,24].

The quality assessment results of the included studies revealed varying levels of bias across both randomized and non-randomized studies. The RoB 2 evaluation for the randomized controlled trials indicated a generally low risk of bias, although some concerns were noted regarding the randomization process in Mody et al. (2017, 2020) [6,21]. In contrast, the ROBINS-I assessment for non-randomized studies demonstrated a serious risk of bias, particularly due to confounding factors and lack of blinding in outcome measurement, as seen in studies such as Raiser et al. (2024) [22], Olgun et al. (2022) [5], and Muñoz et al. (2023) [12]. These findings suggest that while the randomized trials were of relatively high methodological quality, the non-randomized studies had more substantial limitations, primarily related to confounding and measurement bias, which may affect the interpretation of their outcomes. The study quality assessment results are presented in Figure 2.

### 3.2. Baseline Characteristics of Studies

The baseline characteristics of the studies included in the systematic review and meta-analysis are summarized in Table 1. Eight studies were analyzed, comprising both prospective and retrospective designs. The sample sizes ranged from 19 to 146 patients, with age ranges varying significantly between studies. For instance, Olgun et al. (2022) [5] included 19 patients aged between 2.5 and 11 years, while Munoz et al. (2023) [12] featured an older cohort with ages ranging from 1.8 to 33.9 years.

All studies evaluated objective response rate as a primary outcome, while some, such as Mody et al. (2017) [21] and Muñoz et al. (2023) [12], also investigated overall survival and event-free survival. Treatment regimens varied across studies, with most studies incorporating dinutuximab beta in combination with chemotherapy (dB + CT). Median follow-up periods ranged from 10 to 66 months, reflecting variability in monitoring across the studies. Notably, the Mody et al. (2017) [21] and Mody et al. (2020) [6] trials incorporated randomized designs, with the former employing a phase II selection design trial and the latter focusing on a phase II randomized trial investigating combinations such as irinotecan, temozolomide, dinutuximab, and granulocyte-macrophage colony-stimulating factor.

### 3.3. Outcomes of Objective Response Rate (ORR)

**Figure 3:**
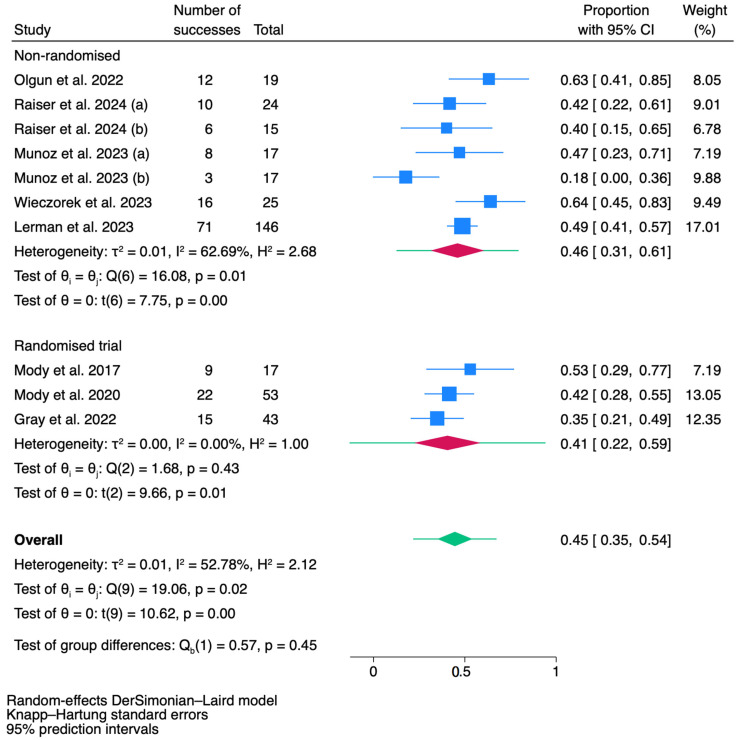
The forest plot of pooled analysis results for overall response rates. CI = confidence interval. Raiser et al. 2024 [22]: a = patients treated by dB + TopoCyclo, b = patients treated with dB + TOTEM/TEMIRI. Munoz et al. 2023 [12]: a = early treatment arm, b = late treatment arm [5,6,7,12,21,22,23,24].

A total of eight studies, comprising ten reports, were included in the pooled meta-analysis for ORR. The overall sample size across these reports was 376 patients. The individual sample sizes ranged from 15 to 146 patients per study arm, reflecting the diversity in patient populations across the included studies. The meta-analysis for the ORR is presented in Figure 3. Across the ten reports, the pooled ORR was 0.45 (95% CI: 0.35–0.54, *p* < 0.001), indicating that approximately 45% of patients demonstrated a favorable treatment response. The individual study ORRs ranged from 0.18 (95% CI: 0.00–0.36) in Muñoz et al. (2023) [12] to 0.64 (95% CI: 0.45–0.83) in Wieczorek et al. (2023) [7]. A random-effects DerSimonian–Laird model was used to account for this variability, and Knapp–Hartung standard errors were applied to ensure robust estimates. The heterogeneity across studies was moderate, with an I^2^ of 52.78% and an H^2^ of 2.12, suggesting some variability in the treatment effects between studies. Funnel plot of the pooled analysis of ORR is presented in Supplementary Figure S1.

The forest plot highlights notable differences between non-randomized studies and randomized trials in terms of heterogeneity and methodological rigor. Observational studies demonstrate moderate heterogeneity (I^2^ = 62.69%, *p* = 0.01), likely reflecting variability in patient populations, treatment protocols, and study designs. In contrast, RCTs exhibit no significant heterogeneity (I^2^ = 0%, *p* = 0.43), indicating a higher degree of consistency across these studies due to stricter protocols and randomization processes. While the pooled proportion for observational studies is slightly higher (0.46 [0.31, 0.61]) compared to RCTs (0.41 [0.22, 0.59]), the difference is not statistically significant (Qb(1) = 0.57, *p* = 0.45).

**Figure 4:**
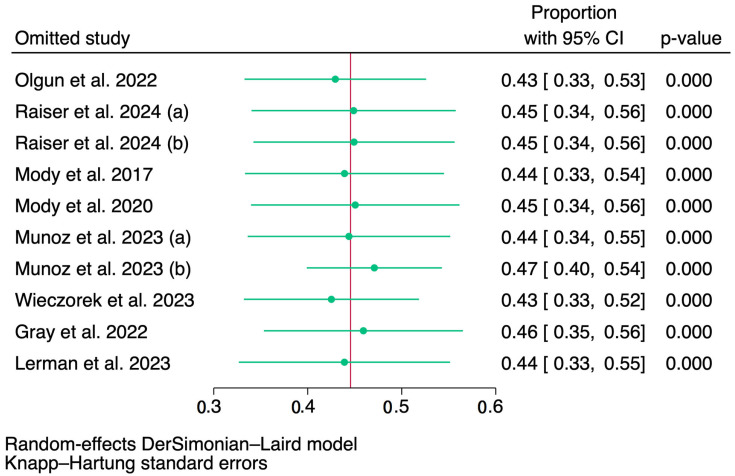
The forest plot of pooled sensitivity analysis results for overall response rates. CI = confidence interval. Raiser et al. 2024 [22]: a = patients treated by dB + TopoCyclo, b = patients treated with dB + TOTEM/TEMIRI. Munoz et al. 2023 [12]: a = early treatment arm, b = late treatment arm [5,6,7,12,21,22,23,24].

Figure 4 illustrates a sensitivity analysis conducted to evaluate the influence of individual studies on the overall effect estimate. Each row represents the exclusion of a specific research. The results demonstrate that the pooled effect estimate remains consistent across all scenarios, suggesting that no single study disproportionately influences the overall outcome. The *p*-values for all analyses remain statistically significant (*p* < 0.05), indicating the robustness of the findings.

### 3.4. Outcomes of Overall Survival (OS)

**Figure 5:**
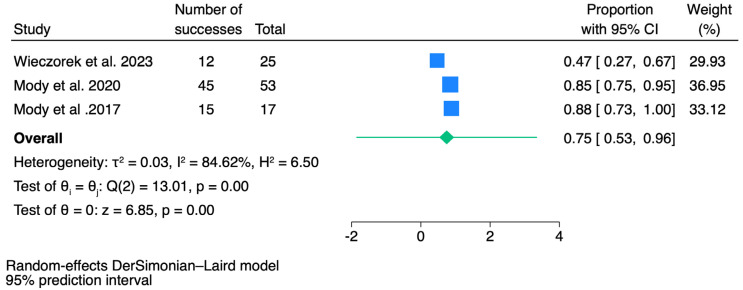
The forest plot of pooled analysis results for overall survival. CI = confidence interval [6,7,21].

For OS, a total of three reports were included in the pooled analysis. As seen in Figure 5, the meta-analysis result was found to be 75% (95% CI: 53–96, *p* < 0.001) for OS. A high level of heterogeneity was found between studies (I^2^ = 84.6%, *p* < 0.001). The highest OS rates were defined as 88% (95% CI: 73–99) [21], and the lowest OS was determined as 47% (95% CI: 27–67) [7].

### 3.5. Outcomes of Event-Free Survival (EFS)

**Figure 6:**
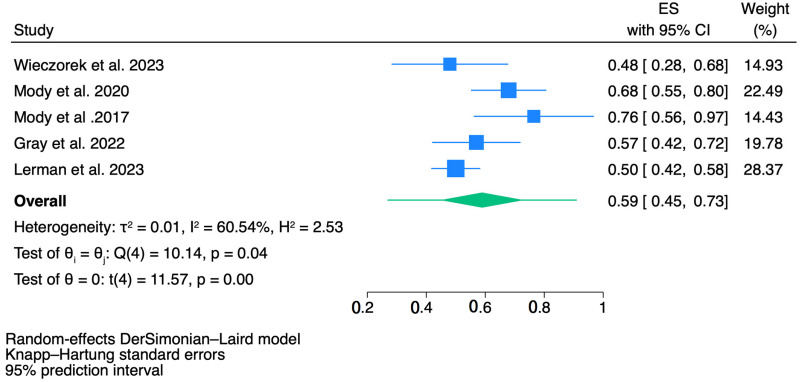
The forest plot of pooled analysis results for EFS scores. CI = confidence interval [6,7,21,23,24].

The meta-analysis for EFS is presented in the forest plot (Figure 6). Across the five included study arms, the pooled EFS was 59% (95% CI: 0.45–0.73, *p* < 0.001). The individual EFS estimates varied across studies, ranging from 48% (95% CI: 0.28–0.68) in Wieczorek et al. (2023) [7] to 76% (95% CI: 0.56–0.97) in Mody et al. (2017) [21].

Significant heterogeneity was observed, with an I^2^ of 60.54% and an H^2^ of 2.53, indicating substantial variability in EFS outcomes across the studies. A random-effects model was applied to account for this heterogeneity, and Knapp–Hartung standard errors were used for improved precision. The test for overall heterogeneity was highly significant (Q(4) = 10.14, *p* = 0.04), reflecting the notable variability in the results.

The studies by Mody et al. (2020) [6] (ES = 0.68, 95% CI: 0.55–0.80) and Mody et al. (2017) [21] (ES = 0.76, 95% CI: 0.56–0.97) reported the highest EFS rates, suggesting the effectiveness of the treatment regimens evaluated in those trials.

## 4. Discussion

This meta-analysis evaluated the efficacy of chemo-immunotherapy with conventional chemotherapy in children with refractory or relapsed neuroblastoma. Our findings indicate that such combinations, particularly involving agents such as dinutuximab, dinutuximab beta, and naxitamab with irinotecan and temozolomide, are associated with a favorable ORR and improved OS and EFS outcomes. The pooled ORR of 0.45 across the included studies highlights the potential of these treatments to induce significant tumor regression in a population where traditional chemotherapy often falls short. These results align with previous research that underscores the role of immunotherapeutic agents, particularly anti-GD2 antibodies such as dinutuximab beta, in enhancing responses when used alongside chemotherapy [9].

Chemo-immunotherapy regimens have emerged as promising approaches for managing refractory or relapsed neuroblastoma, though challenges remain in optimizing efficacy and minimizing toxicity. Studies highlight the use of anti-GD2 antibodies such as dinutuximab and naxitamab combined with chemotherapeutics such as irinotecan and temozolomide, showing notable response rates in patients with high-risk neuroblastoma [6,21,22]. The addition of granulocyte-macrophage colony-stimulating factors (GM-CSF) has been associated with enhanced tumor response and manageable side effects [6]. While promising, outcomes indicate variability in response based on treatment timing and prior therapies, emphasizing the need for personalized and early intervention strategies [12]. Furthermore, the timing and sequencing of these regimens are critical; early salvage therapy with naxitamab-based combinations immediately following induction failure has shown statistically significant improvements in long-term outcomes, including progression-free survival rates after three years [12]. Beyond conventional therapies, research into biomarkers such as killer immunoglobulin-like receptors (KIR) and their ligands highlights the potential to stratify patients and personalize treatments to optimize immune responses [25]. Despite these advances, challenges remain in mitigating toxicity and overcoming resistance. Common toxicities include myelosuppression and pain associated with anti-GD2 therapies, which necessitate careful management and supportive care. The continued exploration of novel agents, such as cyclin-dependent kinase inhibitors and ex vivo expanded NK cells, is paving the way for enhanced efficacy with reduced side effects [26].

Our analysis revealed substantial heterogeneity across the included studies, suggesting variability in patient populations, treatment protocols, and follow-up durations. This heterogeneity underscores the complex biology of neuroblastoma and the differing responses to treatment, which are likely influenced by factors such as tumor genetics (e.g., MYCN amplification), prior treatment exposure, and patient age [27]. Despite these variations, the statistically significant overall effect sizes for both ORR and EFS support the hypothesis that anti-GD2 antibodies [28], particularly when combined with conventional chemotherapy [29], can lead to improved outcomes in this high-risk group of patients.

The use of immunotherapy in several of the included studies, including Mody et al. (2020) [6] and Wieczorek et al. (2023) [7], was associated with higher response rates compared to studies using chemotherapy alone. This is consistent with earlier trials demonstrating that anti-GD2 antibodies enhance the immune system’s ability to recognize and destroy neuroblastoma cells [9]. Furthermore, the combination of dinutuximab with granulocyte-macrophage colony-stimulating factor (GM-CSF) has been shown to stimulate immune effector cells, further boosting anti-tumor activity. These findings are in line with the well-established role of GD2 as a key therapeutic target in neuroblastoma and the growing body of evidence supporting the integration of immunotherapy into standard treatment regimens [10].

Of note, the ORR outcomes of this meta-analysis highlight key findings from both non-randomized and randomized studies, providing insights into the efficacy of treatments for refractory or relapsed neuroblastoma. Non-randomized studies, with a pooled success proportion of 0.46 (95% CI: 0.31–0.61) and moderate heterogeneity (I^2^ = 62.69%), reflect the variability inherent in real-world clinical settings. This heterogeneity likely arises from differences in patient demographics, disease stages, and treatment protocols, underscoring the challenges of standardizing outcomes across diverse study designs. In contrast, randomized trials demonstrate a pooled success proportion of 0.41 (95% CI: 0.22–0.59) with no observed heterogeneity (I^2^ = 0%), showcasing the consistency and reliability afforded by rigorous randomization and controlled methodologies. Importantly, the lack of significant differences between the two study types (Qb = 0.57, *p* = 0.45) supports the broader applicability of the overall pooled estimate (0.45, 95% CI: 0.35–0.54) to inform clinical practice. However, the methodological rigor of randomized trials offers stronger evidence for guiding treatment decisions, while the variability observed in non-randomized studies highlights the need for individualized approaches in heterogeneous patient populations.

While the results of this meta-analysis are promising, there are important considerations regarding the treatment-related toxicities associated with these therapies. The combination of chemotherapy and immunotherapy, particularly anti-GD2 antibodies, has been linked to increased rates of pain, fever, and capillary leak syndrome, which can limit the tolerability of these regimens [9,10,30,31]. Future research should focus on optimizing the balance between efficacy and toxicity, potentially through dosing adjustments, improved supportive care measures, and the identification of biomarkers that can predict which patients are most likely to benefit from these therapies.

The results of the OS meta-analysis, which demonstrated a pooled OS rate of 75%, highlight the variability in survival outcomes for patients with refractory or relapsed neuroblastoma. The high level of heterogeneity observed between studies suggests substantial differences in patient populations, treatment protocols, and follow-up durations across the included studies. This level of heterogeneity is consistent with the findings in the neuroblastoma literature, where factors such as MYCN amplification, age at diagnosis, and prior treatment history are known to significantly impact survival outcomes [27,32]. The highest OS rate, reported at 88%, may reflect more aggressive treatment regimens or a patient cohort with more favorable prognostic factors, whereas the lowest OS rate of 47% likely corresponds to patients with more advanced or refractory disease. These findings align with previous studies that demonstrate a wide range of survival outcomes in neuroblastoma patients based on individual risk factors and treatment responses [33]. Therefore, while the pooled analysis offers a generalized estimate of OS, the wide confidence intervals and significant heterogeneity emphasize the need for more standardized treatment protocols and better identification of prognostic biomarkers to reduce variability in outcomes across clinical trials.

Of note, another important finding of this study is the variability in EFS outcomes across different treatment regimens. The pooled EFS of 0.59 highlights the potential for prolonged disease control, yet the heterogeneity in outcomes suggests that some patients may derive more benefit from specific combinations. Studies such as Mody et al. (2017) [21], which reported the highest EFS rates, underscore the importance of selecting the right combination of agents to maximize long-term disease control. The inclusion of irinotecan and temozolomide, alongside dinutuximab beta, in several studies further supports the concept that multi-agent chemotherapy combined with immunotherapy may be necessary to achieve optimal outcomes [34].

Neuroblastoma is a complex pediatric malignancy that often presents substantial therapeutic challenges, particularly in refractory or relapsed settings. Personalized treatment strategies offer a promising avenue to improve outcomes; however, current evidence and guidelines on how best to incorporate biomarkers remain insufficiently detailed. Future investigations should prioritize the identification and validation of clinically actionable markers, including genetic mutations, epigenetic alterations, and immunological features (e.g., circulating tumor DNA or tumor-infiltrating lymphocytes) [35]. By stratifying patients based on distinct molecular or immunological signatures, clinical trials could be designed to evaluate targeted agents or immunotherapies in more homogeneous subgroups [36]. Furthermore, the increasing adoption of adaptive trial designs presents an opportunity to efficiently assess multiple therapeutic strategies in parallel [37]. Such approaches not only enhance patient selection but also facilitate real-time data analysis and protocol adjustments, potentially accelerating the discovery of optimal chemo-immunotherapy regimens for specific neuroblastoma populations [38].

Toxicity remains a considerable obstacle to the broader application of chemo-immunotherapy protocols for pediatric patients with advanced disease. Although recognized as a major limitation, further exploration of mitigation strategies is warranted, including alternative dosing schedules and advances in supportive care. Metronomic chemotherapy, which involves administering lower, more frequent doses, has shown potential to reduce toxicity without compromising efficacy in pediatric populations [39]. Concurrently, developments in pharmacogenetics could enable clinicians to tailor both dosing and regimen selection to each patient’s unique genetic profile, thereby minimizing severe adverse events [40]. Additionally, early implementation of proactive supportive care—such as comprehensive antiemetic regimens, growth factor support, and immunomodulatory adjuncts—can help sustain treatment adherence while enhancing overall quality of life [41]. Future trials that incorporate robust toxicity endpoints are essential for evidence-based recommendations, ensuring that efficacy is balanced with acceptable safety profiles in the management of relapsed or refractory neuroblastoma [36].

Of note, the risk of bias analysis conducted using the RoB 2 and ROBINS-I tools highlights significant variability in the methodological rigor of the included studies, which has implications for the interpretation of the results. For randomized trials (Figure 2A), the overall risk of bias was categorized as “some concerns”, primarily due to issues related to deviations from intended interventions (D2) and the selection of reported results (D5). While randomization processes were generally robust, concerns in these domains suggest that certain trials may have experienced deviations that could influence the reliability of outcome measures. In contrast, the non-randomized studies (Figure 2B) demonstrated a higher overall risk of bias, with “moderate” to “serious” ratings across several domains, particularly confounding (D1), selection bias (D2), and classification of interventions (D3). This reflects the inherent challenges of non-randomized study designs, such as the lack of randomization and the potential for unmeasured confounders. These biases may contribute to the heterogeneity observed in pooled estimates and should be carefully considered when interpreting the findings.

This meta-analysis has several limitations that must be acknowledged. First and foremost, the number of included studies was relatively small, which limits the generalizability of our findings. Although the studies analyzed provided valuable insights into the efficacy of anti-GD2 antibodies combined with conventional chemotherapy, the limited sample sizes and the small number of available trials may reduce the statistical power of the analysis. This is particularly important given the complexity of neuroblastoma as a disease and the potential variability in response to treatment across different patient populations. Furthermore, there were significant differences in the design and methodology of the included studies, which introduce heterogeneity into the analysis. The meta-analysis incorporated both RCTs and non-randomized studies, such as retrospective analyses and single-arm clinical trials. These differences in study design could influence the outcomes, as RCTs generally offer a higher level of evidence compared to observational studies. Additionally, the inclusion of studies with varying follow-up durations and treatment regimens adds to the complexity and makes direct comparisons between studies more challenging. This heterogeneity was reflected in the I^2^ values, particularly for EFS-, which showed substantial variability across studies. Taken together, there were differences in the patient populations, such as age ranges and prior treatments, which could affect the results. Some studies included older patients or those with more heavily pretreated disease, which could impact their ability to respond to further therapies. Future research should focus on conducting larger, multicenter randomized trials to provide more robust evidence on the efficacy of these combination therapies in children with refractory or relapsed neuroblastoma.

The most important limitation of this meta-analysis is the notable variability observed in ORR, OS, and EFS outcomes across the included studies. While this variability may be influenced by differences in patient demographics (e.g., age, disease stage, prior treatments) and treatment protocols (e.g., chemotherapy regimens, dosing schedules, supportive care measures), the available data did not allow for a comprehensive analysis to fully explore these potential sources of heterogeneity. Despite this, the pooled estimates provide valuable insights, and future studies with more detailed and consistent reporting may help to better understand and address these variations.

Another limitation of this meta-analysis is the inability to fully account for potential confounding variables, such as prior treatments or genetic differences, which may have influenced the outcomes. While quality and bias were systematically assessed, variations in baseline characteristics, including treatment history and patient-specific genetic profiles, could not be thoroughly examined due to the limitations in the primary study reports. These constraints, inherent to aggregate data meta-analyses, may have contributed to the observed heterogeneity and limited the capacity to explore these factors in depth. Future research would benefit from individual patient data (IPD) meta-analyses, which would allow for a more detailed investigation of the impact of confounding variables and provide a more robust understanding of treatment effectiveness across diverse patient subgroups.

## 5. Conclusions

In conclusion, this meta-analysis highlights the promising potential of combining anti-GD2 antibodies with conventional chemotherapy in the treatment of children with refractory or relapsed neuroblastoma. Our findings demonstrate that these combinations, particularly involving agents such as anti-GD2 antibodies, significantly enhance objective response rates and extend event-free survival, offering new hope for a population with historically poor outcomes. However, the variability in study designs and patient populations underscores the need for more robust, standardized research to fully unlock the potential of these therapies. Looking forward, future studies must prioritize large-scale, multicenter randomized controlled trials that not only confirm the efficacy of these regimens but also optimize their safety and tolerability. Furthermore, the development of predictive biomarkers will be essential in tailoring treatments to individual patients, ensuring that the most effective therapies are delivered to those most likely to benefit. By addressing these critical gaps, the next generation of research can pave the way for more personalized, precise, and effective treatments for one of the most challenging pediatric cancers. Chemo-immunotherapy regimens have shown substantial promise in treating refractory or relapsed neuroblastoma, particularly when tailored to individual patient profiles and initiated early in the treatment trajectory. Ongoing research and biomarker-driven strategies will be pivotal in further improving outcomes.

## Figures and Tables

**Table 1:** Baseline characteristics of studies included in the systematic review and meta-analysis.

First Author/Year	Study Type	Sample Size (*n*)	Age (Range), Years	Event of Death (*n*)	Type of Treatment	Follow-Up Time (Range), Months	Outcome of Interest
Olgun et al. 2022 [5]	Retrospective analysis of prospectively collected data	19	5.5 (2.5–11)	N/A	dB + CT	11 (6–26)	ORR
Raiser et al. 2024 [22]	Prospective non-interventional study	39	5.5 (1–24)	N/A	dB + TopoCyclo	10	ORR
Mody et al. 2017 [21]	Randomized Phase II selection design trial	35	5.7 (2.1–16.2)	14	dinutuximab+ granulocyte-macrophage colony-stimulating factor	25	ORR, OS, EFS
Mody et al. 2020 [6]	Randomized phase II trial	53	3.2 (1.7–11.9)	16	irinotecan, temozolomide, dinutuximab, and granulocyte-macrophage colony-stimulating factor	19.2 (14.4–51.6)	ORR, OS, EFS
Muñoz et al. 2023 [12]	Retrospective analysis of prospectively collected data	34	4.9 (1.8–33.9)	N/A	irinotecan, temozolomide, and naxitamab plus GM-CSF (HITS)	26.9 (2.8–57.9)	ORR
Wieczorek et al. 2023 [7]	Retrospective analysis of prospectively collected data	25	2.9 (0.5–8.3)	N/A	dB + CT	47	ORR, OS, EFS
Lerman et al. 2023 [24]	Retrospective study	146	4.25 (2.5–6)	N/A	irinotecan/temozolomide/dinutuximab/granulocyte-macrophage colony-stimulating factor	66	ORR, EFS
Gray et al. 2022 [23]	Randomized phase II trial	65	4 (1–21)	N/A	dB + CT	18	ORR, EFS

N/A: not available, ORR: objective response rate, OS: overall survival, EFS: event-free survival, dB: dinutuximab beta, CT: conventional chemotherapy.

## Data Availability

The datasets used and/or analyzed in this study are available upon reasonable request from the corresponding author.

## Supplementary Materials

The following supporting information can be downloaded at: https://www.mdpi.com/article/10.3390/jcm14030934/s1. Table S1: The PRISMA checklist; Table S2: Search strategy used for the systematic review in PubMed, EMBASE, SCOPUS, and WoS electronic databases; Figure S1: The funnel plot of pooled analysis results for overall response rates; Figure S2: The funnel plot of pooled analysis results for overall survival; Figure S3: The funnel plot of pooled analysis results for event-free survival; Supplementary Materials File S1: Additional information about the trials.
